# The Second Silent Pandemic: Why Arboviruses Demand an Orchestrated Global Health Response

**DOI:** 10.3390/pathogens15040398

**Published:** 2026-04-07

**Authors:** Nguyen Khoi Quan, Andrew W. Taylor-Robinson

**Affiliations:** 1 College of Health Sciences, VinUniversity, Hanoi 100000, Vietnam; quan.nk@vinuni.edu.vn; 2 Center for Innovations in Health Sciences, VinUniversity, Hanoi 100000, Vietnam; 3 Johns Hopkins Bloomberg School of Public Health, Johns Hopkins University, Baltimore, MD 21205, USA; 4 Center for Global Health, Perelman School of Medicine, University of Pennsylvania, Philadelphia, PA 19104, USA

**Keywords:** arbovirus, mosquito, global health, epidemic, climate change, urbanization

## Abstract

Infections caused by arboviruses, a diverse group of viral pathogens transmitted by biting arthropod vectors, mainly mosquitoes, ticks, and midges, can cause a range of illnesses in humans, from mild, influenza-like symptoms to severe neurological complications including encephalitis and viral hemorrhagic fever. According to 2024 World Health Organization statistics, vector-borne diseases collectively account for over 700,000 human deaths annually, with mosquito-borne infections such as dengue, chikungunya, Zika, and yellow fever constituting a growing and significant proportion of this burden. What was once considered a problem localized to poorly resourced settings in tropical and subtropical regions is now becoming a pervasive global challenge. This is due largely to a combination of factors including climate change, transcontinental travel, and urbanization, with the geographical spread and intensity of arboviral outbreaks reaching unprecedented levels during the current century. In much the same way that the escalating global burden of bacterial infections resistant to antibiotics has been described as a silent pandemic, the insidious rise of arboviruses begs questions regarding outbreak preparedness, prevention and control. Here, we highlight the pressing need for comprehensive strategies that incorporate various health sectors to mitigate the emergence and resurgence of arboviral diseases. Future directives that should be prioritized are outlined. As demonstrated by epidemiological trends and historical outbreak data, an orchestrated global response is critical not only for managing current threats but also for preventing future epidemics.

## 1. Introduction

An arbovirus (short for *ar*thropod-*bo*rne virus) is one of a taxonomically heterogeneous array of viral pathogens transmitted to human and animal hosts primarily through blood-feeding arthropod vectors such as mosquitoes, ticks, sandflies, and midges. Infection with such significant pathogens as dengue, chikungunya, Zika, and yellow fever viruses may cause clinically serious conditions including encephalitis, hemorrhagic fever and systemic febrile illnesses, which collectively represent a rapidly escalating crisis for global health security. While historically confined to the tropics, the geographical and epidemiological boundaries of these mostly positive-sense RNA viruses are breaking down [1,2]. Governed largely by transmission via the highly adaptable *Aedes aegypti* and *Ae. albopictus* mosquitoes, arboviruses are no longer just a localized threat; they are responsible for a substantial and increasing share of global infectious disease morbidity, with dengue serving as the primary exemplar [3,4]. While mortality rates are markedly lower than for the *Anopheles* mosquito-borne parasitic disease malaria, the true threat of arboviruses lies in their capacity to paralyze healthcare systems and economies through explosive outbreaks, severe neurological anomalies like microcephaly, and long-term disabilities [4,5]. Factors like climate change, increased globalization, and urbanization exacerbate the geographical spread and intensity of outbreaks of these diseases [4,6,7,8,9].

All species of mosquito, the principal conduit of vector-borne diseases, require standing water in which to breed. In the context of mosquito dynamics, the clearest impact of climate change is rising temperatures and altered intensity, temporal and spatial patterns of rainfall [10]. In tropical and subtropical zones this may cause a shifting monsoon season, thus affecting directly the distribution of the invertebrate vector and indirectly that of the pathogen it transmits. The outcome can also be a more dramatic meteorological event like a tropical cyclone. Such disasters can severely disrupt public health infrastructure, greatly limit access to prevention and treatment programs, and substantially increase infection risk, especially in high-burden regions where continuity of care and control is critical [11]. Climate change may lead to significant shifts in the habitat suitability for mosquito vector propagation, with implications for the transmission dynamics and distribution of arboviruses [10,12]. However, despite these collective concerns, longitudinal, intervention-level data quantifying how specific climate-related disruptions—such as altered rainfall seasonality or post-cyclone habitat expansion—translate into measurable changes in dengue vector control efficacy remain scarce.

The escalation of arboviral infections reflects changes in vector behaviors and environmental factors conducive to their transmission. Urbanization and population growth create ideal conditions for mosquitoes to thrive, particularly in tropical and subtropical regions where these viruses are endemic. The past few decades have seen a surge in arboviral activity, with one modeling estimate indicating dengue virus infects close to 400 million people annually, of whom one quarter experience symptomatic illness [13]. Moreover, another study on the prevalence of dengue estimates that 3.9 billion people are at risk of infection [4]. The Zika virus outbreak that emerged in 2015 spurred global awareness due to its association with severe neurological disorders in infants, elevating the level of urgency regarding arboviral diseases [5,7].

As arboviruses expand their geographical range, the potential for new outbreaks heightens, partly propelled by increased movement of people and goods across borders. Therefore, a multifaceted approach grounded in the ‘One Health’ perspective is vital [14]. This emphasizes collaboration across sectors to address the interconnectedness between human, animal, and environmental health, crucial for mitigating these risks effectively [15]. Without coordinated international efforts and robust surveillance and control measures, the public health threat posed by arboviruses is likely to escalate further. In coining the term ‘second silent pandemic’ we draw explicit analogy with the widely recognized, so-called silent pandemic of antimicrobial resistance that is illustrated primarily by a reduced susceptibility of pathogenic bacteria to previously effective antibiotics [16]. These are distinct infectious threats, yet they share a cross-border escalation that unfolds below the threshold of general public awareness (so, very different to the coronavirus disease (COVID)-19 pandemic), each rising insidiously to a level that is causing a catastrophic global health impact.

## 2. Dengue: A Harbinger of Expansion

The dramatic expansion of dengue serves as a stark example of the evolving threat posed by arboviruses. Initially confined to just nine countries in the 1970s, dengue has now infiltrated over 140 nations globally [13]. Beyond the annual death rate of approaching 30,000, the socioeconomic burden may be gauged by considering a loss of 2.08 million disability-adjusted life-years (DALYs) in 2021 [17]. The emergence of dengue has not followed a gradual increase, as reported incidence has surged ten-fold so far this century, significantly affecting regions that were previously considered non-endemic [18]. The spread of dengue is driven by a multifaceted interplay of factors including accelerated rates of global travel, urbanization, deforestation, and climate change, which create favorable conditions for both the dengue virus and its mosquito vectors to thrive in new environments [6].

As climate change improves habitat suitability and expands ranges of most *Aedes* vector species, mitigating future climate change will be a key approach to lessen their impacts [19]. Urbanization similarly elevates the risk, as densely populated areas provide breeding grounds for peridomestic *Aedes* spp., which are the primary vectors for dengue transmission [6]. Furthermore, high rates of international travel have raised considerably the potential for imported cases to regions that lack robust surveillance and vector-control measures, compounding the risk of local transmission [1].

The complex factors leading to the surge in dengue cases highlight a public health challenge that exceeds the traditional bounds of surveillance and response. The increasing number of dengue infections emphasizes the need for systemic changes and coordinated global strategies to combat the threats posed by arboviral diseases [6]. The holistic ‘One Health’ concept, integrating people, animals, and environment, is essential for addressing the underlying conditions facilitating such outbreaks [8].

## 3. The Emergence and Re-Emergence of Other Arboviruses

The recent period of increased dengue incidence has been paralleled by the emergence and re-emergence of other arboviral diseases of public health concern. Chikungunya, for instance, has caused explosive outbreaks following a critical mutation in the virus envelope glycoprotein, characterized as E1:A226V, identified in 2005 [20]. This enhances the ability of the virus to infect *Ae. albopictus* mosquitoes and has contributed to its geographical spread, resulting in widespread epidemics in previously unaffected populations [20,21]. The introduction of chikungunya viral strains carrying this mutation has transformed it from a localized threat into a global health concern, with numerous outbreaks reported across several continents, particularly affecting naive populations [21].

Other mosquito-borne diseases, such as yellow fever, which have historically been more geographically and temporally contained, continue to pose risks for large urban outbreaks. Particularly vulnerable populations with limited access to healthcare and inadequate vector control measures still maintain a high potential for significant outbreaks [22]. Urbanization and the movement of susceptible populations contribute to the risk of vector-borne disease transmission, including yellow fever outbreaks in cities with *Aedes* mosquito populations [22,23].

Aside from the aforementioned arboviruses that currently pose a global threat, there are many more obscure arboviruses of regional public health concern but about which considerable gaps in epidemiological knowledge exist. For instance, over 75 arboviruses have been identified that are indigenous to Australia, the most important of which from a clinical perspective are Ross River, Barmah Forest, Murray Valley encephalitis, and West Nile (Kunjin strain) viruses [24]. While the transcontinental spread of Zika within the last decade offers a precedent, it remains uncertain whether any specific understudied Australian arbovirus could achieve comparable international reach; nonetheless, this possibility warrants proactive surveillance [25]. In Latin America, the same principle applies to Mayaro and Oropouche viruses, while Usutu virus has spread from southern Africa to Europe [26,27,28]. Due to a combination of non-specific signs and symptoms of undifferentiated febrile illness, limited or no availability of diagnostic tests, frequent lack of identification of vectors, and a fragmented understanding of transmission dynamics, the prevalence in the human population for each of these neglected causal agents of viral infection is likely grossly underestimated [24,29].

The interconnected nature of arboviral threats in humans and animals has significant implications for disease surveillance and control strategies. Active surveillance for one arbovirus often uncovers co-circulating and sometimes related viruses. This phenomenon highlights the necessity for robust, integrated surveillance frameworks that monitor multiple pathogens concurrently, improving responsiveness and resource allocation in public health systems [29]. Collaborative efforts to enhance surveillance can facilitate early detection and response, enhance interventions, and thereby mitigate the impacts of arboviral disease outbreaks.

## 4. The Expanding Global Risk Landscape

Currently, almost half of the world’s population is at risk of dengue, a figure that showcases the widespread reach of this arboviral disease [4]. Particularly concerning is the rise in autochthonous cases—locally acquired infections—reported in regions that were previously considered non-endemic, indicating a worrying proliferation of arboviruses [21]. Factors driving this escalating risk are complex and interconnected with modern global trends. Among European territories, for instance, a notable dengue outbreak in Madeira (an autonomous region of Portugal located in the Atlantic Ocean) in 2012–2013 resulted in over 2000 locally acquired cases [30], while entomological surveillance confirmed the establishment of *Ae. albopictus* and detection of *Ae. aegypti* in Cyprus, in the eastern Mediterranean Sea [31], underscoring that no region can consider itself permanently non-endemic.

Migration is a significant factor, facilitating the mobility of both infected individuals and vectors across borders. As people travel and settle in new areas, they can inadvertently introduce arboviruses and competent mosquito vectors into regions that lack a history of exposure [6]. Globalization further compounds this risk through increased trade and travel patterns that enable viruses to infiltrate new territories more easily [6]. Urbanization also plays a crucial role; densely populated urban areas often face inadequate sanitation measures, creating prime breeding grounds for mosquitoes like *Ae. aegypti*, the primary vector for dengue [6,32]. The increased interaction between humans and mosquitoes in these settings enhances the likelihood of disease transmission [32]. This reflects a ‘paradox of progress’ in rapidly developing nations like Vietnam, where significant economic growth and infrastructure development have inadvertently created new social determinants of infectious disease transmission, facilitating the urban spread of *Aedes*-borne pathogens despite overall improvements in healthcare access [33].

The growing impact of anthropogenic influences on Earth’s climate systems is alarming. Global warming and other key indicators of extreme weather point to climate change as a pivotal factor influencing the spread of arboviral diseases [3]. Alterations in surface temperature and rainfall patterns are expanding the geographical range suitable for *Aedes* spp. mosquito vectors, impacting their life cycles and the replication rates of the viruses they carry [3]. Modelling projections suggest that warming temperatures could expose an additional 1.3 billion people to the risk of diseases like dengue and Zika by 2050, highlighting climate change as both a direct and indirect driver of arboviral disease dynamics [10,34].

## 5. Complexities in Disease Management and Surveillance

The fight against arboviruses, particularly dengue, continues to face substantial challenges despite innovative therapeutics and advancements in vector control solutions. One of the primary complexities of dengue management stems from the circulation of four related but distinct dengue virus serotypes (DENV-1, -2, -3, and -4) [35]. While infection with one serotype confers lifelong immunity to that specific serotype, it only provides temporary and partial immunity to other serotypes. Consequently, subsequent infections with a different serotype can increase the risk of severe disease outcomes, such as dengue hemorrhagic fever and dengue shock syndrome [35,36]. The ongoing circulation of these serotypes creates a public health conundrum, necessitating careful monitoring and management strategies to mitigate the risk of severe complications following secondary infections [36]. A key biological basis for this heightened severity is antibody-dependent enhancement, whereby non-neutralizing antibodies from a prior infection can, paradoxically, facilitate viral entry into monocytes during a subsequent heterotypic infection, amplifying viremia and inflammatory responses [37]. This phenomenon, compounded by the existence of four recognized, antigenically distinct serotypes, presents a formidable barrier to dengue vaccine development: any vaccine must confer balanced, durable protection against all four serotypes simultaneously in order to avoid inadvertently priming recipients for enhanced manifestations of disease [36,37].

Compounding this issue is the clinical overlap that arboviral diseases present, often featuring similar, non-specific symptoms. This can lead to misdiagnoses and subsequent misreporting of cases [21]. Symptoms of dengue, chikungunya, and other mosquito-borne diseases can be indistinguishable, contributing to a considerable burden on healthcare systems due to diagnostic inaccuracies [21,38]. Furthermore, inadequate active surveillance for febrile illnesses exacerbates these challenges, hindering accurate outbreak monitoring and timely implementation of necessary control measures [39]. The lack of precise data on dengue and related arboviral diseases complicates the allocation of resources and effective response strategies, thereby limiting the capacity to evaluate the impact of public health interventions [39].

An integrated approach that encompasses both laboratory diagnostics and clinical assessments, inclusive of the nuanced understanding of serotype variation and the potential for co-infections, is necessary for effective patient monitoring [40]. The ability to accurately diagnose and differentiate between various arboviral infections, as well as to distinguish from other infectious diseases with partly overlapping clinical symptoms, will enhance outbreak management and thereby ultimately contribute to more effective prevention strategies [40,41]. These diagnostic challenges are not limited to leading arboviral infections like dengue, chikungunya, Zika, and yellow fever. Rather, they represent a systemic vulnerability that applies equally across the broader landscape of arboviral human pathogens, where clinical heterogeneity and limited point-of-care testing capacity compound surveillance deficits in endemic and emerging non-endemic regions [42].

## 6. Fragmented Responses to a Globalized Threat

Despite the global nature of arboviruses and the interrelated forces driving their expansion, the response to these outbreaks frequently remains fragmented. The responsibility for managing arboviral outbreaks often falls under the remit of individual national health authorities, with local and municipal agencies typically tasked with instigating early warning systems for controlling disease-carrying mosquito vectors [2]. This decentralized approach, while understandable in terms of local autonomy, proves to be largely ineffective against a threat that crosses national borders without regard for jurisdictional constraints [21].

Such decentralization of response is underscored by significant gaps in diagnosis and surveillance capabilities [29]. The clinical manifestations of arboviral diseases often overlap, leading to misdiagnoses and data inconsistencies that severely hinder accurate outbreak monitoring [38]. The insufficiency of active surveillance and the reliance on local entities to manage vector control contribute to these ongoing challenges. Consequently, this fragmentation complicates the implementation of timely and effective control measures, which is essential for managing public health initiatives [29].

The lessons learned from the disjointed response to the recent COVID-19 pandemic highlight the critical need for integrated global action in predicting, preparing for, and reacting to health crises. The pandemic underscored the importance of coordinated efforts, where effective surveillance systems and comprehensive strategies could significantly enhance the response to outbreaks of infectious diseases [15]. However, consensus on precisely how this should be achieved is still to be reached among the global health community. These insights should serve as an impetus for change, urging health authorities to adopt more unified approaches to arboviral disease management that involve cooperation across borders and sectors, consequently improving preparedness and response strategies [8,15].

Moving towards a more integrated and collaborative framework for surveillance and management can help to address the existing gaps in response strategies related to arboviral diseases. By learning from past experiences and recognizing the interconnected nature of these threats, it is possible to create a more effective and responsive global health system [8].

## 7. Towards a Coordinated Global Strategy

The rapidly growing threat of arboviruses and the currently fragmented response to it urgently demands improved international cooperation and collaboration across diverse fields. The need for a coordinated global strategy is paramount. Of these, surveillance interoperability and laboratory network integration represent the foundational prerequisites upon which equitable procurement, community health systems, and artificial intelligence-assisted prediction can meaningfully build. First, the development of robust and standardized dengue surveillance systems is crucial [29]. These systems should be interconnected across countries, enabling real-time data sharing and rapid response during outbreaks. Laboratory confirmation of dengue cases improves the specificity of surveillance data and can be achieved through national and international networking of laboratories. This facilitates sharing expertise, protocols, and data essential for effective surveillance [39].

Furthermore, coordinated procurement of innovative vector control strategies, treatments, and vaccines is vital to ensuring equitable access and effective deployment, particularly in resource-limited settings [21]. Optimized surveillance systems can significantly reduce the economic burden of outbreaks by facilitating timely responses. As one illustrative, regionally specific estimate, in Australia a delayed response to dengue outbreaks of 4–6 weeks can lead to financial costs more than one hundred times higher than when active surveillance is instigated within two weeks [39]. As demonstrated in Nepal, primary healthcare centers and community volunteers play a key role in epidemiological surveillance and management, indicating that a multi-faceted approach involving various healthcare levels is necessary for effective outbreak preparedness and response [43].

Additionally, international collaboration is essential for expediting the research and development of new tools to control arboviruses. Sovereign states, dependencies and territories need to work together to understand the complex epidemiology of these diseases, which often requires cross-border data sharing and collaborative research initiatives [21]. Educating community workers and training healthcare professionals in affected regions becomes imperative to equip them with the skills and resources necessary to respond effectively to arboviral outbreaks [44]. Artificial intelligence and machine learning offer an expanding toolkit for outbreak prediction and diagnostic screening [42]—roles already being piloted in resource-limited settings [45]—although their deployment must be paired with the underlying data infrastructure and human capacity to interpret and act on model outputs.

The challenge presented by arboviruses is now fundamentally worldwide as a result of a climate-driven changing environment and shifting land use patterns, thus highlighting the necessity of a unified and sustainable approach to enhance global preparedness and response strategies [6]. Utilizing lessons learned from recent global health crises, such as the COVID-19 pandemic, reinforces the importance of coordinated efforts to protect public health. By fostering international collaboration that integrates various stakeholders, including governments, healthcare systems, and at-risk populations, the global health community can enhance its capability to pre-emptively manage and respond to the evolving threat of arboviruses [8].

## 8. Future Directives

Addressing the escalating arboviral threat requires a transition from abstract global goals to localized, functional infrastructure. Future directives must prioritize the following pragmatic pillars to ensure resilience in resource-constrained settings. These directives represent the authors’ synthesis of evidence-based priorities drawn from the literature reviewed above, rather than providing formal consensus guidelines; they are intended to stimulate structured debate and policy consideration:

*Front-Line Diagnostic Equity and Multiplexing*: Priority must shift toward the mass distribution of low-cost, shelf-stable rapid diagnostic tests at the point of care. Surveillance systems should move away from single-virus monitoring in favor of multiplex panels that screen for a “febrile bundle” (e.g., dengue, chikungunya, and Zika) [46]. This enables immediate clinical management and transforms surveillance from a retrospective exercise into a predictive tool, addressing the clinical overlap and misdiagnosis common among these often co-circulating viruses [38].

*Unified One Health Governance*: Effective implementation requires coordinated budgetary planning and, where politically feasible, joint funding mechanisms between government ministries of health, agriculture, and the environment. Establishing unified emergency funds ensures that the detection of a virus in animal host or vector populations triggers a funded response before significant human transmission occurs. This multisectoral approach is critical for defining inter-sectoral priorities and bridging data gaps that currently hinder accurate risk assessment [8,15].

*Infrastructure-Led Vector Control*: While innovative vector control solutions involving genetic modification of mosquitoes, sterile insect techniques, and the wider deployment of *Wolbachia*-infected mosquitoes offer promise for reducing viral transmission [47], immediate focus must remain on basic urban engineering. Improving piped water reliability and waste management eliminates the man-made breeding habitats that enable urban proliferation [32]. Vector control must be integrated with climate-sensitive town and city planning to mitigate the impacts of rapid urbanization and migration, bridging landscape ecology with urban science [2,32].

*Decentralized Micro-Surveillance*: Surveillance should be rooted in community engagement and education to ensure successful adoption and long-term sustainability. Training local leaders to report “unusual clusters” via a smartphone app or web-based dashboard provides high-sensitivity “human intelligence” [48], which serves as the primary data feed for advanced AI and machine-learning predictive models [42,45]. These digital interventions can empower communities to actively participate in vector control and early warning systems.

*De-politicized Data and Procurement*: International treaties are needed to protect the sharing of real-time epidemiological data across borders without the threat of economic sanctions. Furthermore, regions must establish pre-negotiated procurement contracts to ensure equitable access to vaccines and medical supplies at fixed costs, preventing the supply chain failures observed in previous pandemics [49]. Strengthening these mechanisms is essential for global health security and bridging gaps in surveillance equity [29].

*Vaccine Progress*: Theoretically, at least two licensed dengue vaccines are currently available: CYD-TDV (Dengvaxia), which is recommended by the World Health Organization strictly for seropositive individuals aged 9–45 years in endemic settings to avert antibody-dependent enhancement [50]; and TAK-003 (Qdenga), built on a DENV-2 backbone, which does not require individual pre-vaccination serological screening [51]. Unfortunately, Sanofi-Pasteur has ceased production due to what it claims is a lack of global demand. Yellow fever remains preventable with the highly efficacious live-attenuated 17D vaccine [52]. In addition, the chikungunya prophylactic landscape has recently evolved; following the Food and Drug Administration’s August 2025 US market suspension and subsequent January 2026 withdrawal of the live-attenuated Ixchiq vaccine due to safety concerns, the virus-like particle vaccine VimKunya serves as the primary licensed alternative for people from 12 years and older at increased risk of exposure [53,54]. Integrating one or more of these vaccines into national immunization programs represents a critical, underutilized facet of arboviral disease control. Looking long-term, sustained investment is required to research and develop both pan-arbovirus vaccines and broad-spectrum antivirals [21,55].

*Improved Awareness in Non-Endemic Settings*: Preventive approaches must be paired with strengthened healthcare infrastructure and specialized training for clinicians to recognize diverse arboviral presentations in emerging non-endemic regions. Diagnostic accuracy in primary care in low-resource settings is currently low [38], and will remain so without efforts to provide continuous professional education and laboratory support.

## 9. Discussion

Arboviruses present a rapidly intensifying global health emergency. The dramatic increase in the prevalence of dengue, coupled with the emergence and re-emergence of other pathogenic arboviruses like chikungunya, Zika and yellow fever, paints a clear picture of a threat that is both intensifying and expanding geographically [21]. Drivers such as globalization, urbanization, migration, and climate change are inexorably widening the reach of these diseases, placing billions more people at risk [6,10]. In light of this insidious threat—a “phantom menace” hiding in plain sight—urgent and orchestrated action is needed to avoid a global health catastrophe in coming decades, much like the call to arms regarding the silent pandemic burden of antimicrobial resistance.

Scientific advancements and technological innovations provide a beacon of hope amidst these multifactorial, interrelated challenges. However, the current fragmented global response, characterized by insufficient surveillance and decentralized control efforts, severely limits our capacity to combat these pathogens effectively [21]. Analysis indicates that increased outreach for integrated surveillance systems can greatly enhance detection capabilities, thereby addressing the gaps in real-time monitoring of arboviral diseases [29].

The recent COVID-19 pandemic offers crucial lessons, particularly emphasizing the importance of international collaboration and a unified approach to global health threats. For arboviruses, this translates into an immediate necessity for robust, integrated surveillance systems that utilize advanced technology for predictive modeling of outbreaks [14,29]. Alongside this, there is a pressing need for accelerated research and development of new or improved diagnostics, treatments, and vaccines. Coordinated deployment of innovative vector control strategies is also essential, particularly in adapting to the evolving challenges to human health posed by these infectious agents [2].

## 10. Conclusions

The evolution of dengue from a localized health issue to a global epidemic, seemingly by stealth since the second half of last century, illustrates the critical need for enhanced preparedness and effective public health interventions to adapt to the complex and changing landscape of arboviral diseases. The interconnectedness of migration, globalization, urbanization, and climate change heightens the risk of arboviral diseases and emphasizes the broad challenges public health systems face in managing and mitigating the associated risks. Integrated surveillance and control strategies must consider the multifaceted nature of arboviral outbreaks, ensuring that public health responses can adapt effectively to this evolving and complex landscape.

Moving forward, a paradigm shift is required—from reactive, localized responses to proactive, globally orchestrated initiatives that encompass multifaceted strategies across scientific, public health, and policy domains. Only through sustained international cooperation, data sharing, and active research can we hope to mitigate the growing menace of arboviruses and safeguard global health for future generations.

## Data Availability

No new data were created or analyzed in this study. Data sharing is not applicable to this article.
